# Prevalencia del síndrome de *burnout* en médicos familiares que laboran en Colombia y factores asociados

**DOI:** 10.15446/rsap.V25n5.100694

**Published:** 2023-09-01

**Authors:** Jorge L. Ramirez-Rios, Juan F. Pardo-Báez, Cesar J. Fuentes-Estepa, José D. Toledo-Arenas

**Affiliations:** 1 JR: MD. Esp. Medicina Familiar. Esp. Epidemiología. Esp. Seguridad y Salud en el Trabajo. Universidad El Bosque. Bogotá, Colombia. jlramirezr@unbosque.edu.co Universidad El Bosque Universidad El Bosque Bogotá Colombia jlramirezr@unbosque.edu.co; 2 JP: MD. Esp. Medicina Familiar. Esp. Auditoría Medica. Esp. Gerencia en Seguridad y Salud en el Trabajo. Universidad El Bosque. Bogotá, Colombia. jfpardob@unbosque.edu.co Universidad El Bosque Universidad El Bosque Bogotá Colombia jfpardob@unbosque.edu.co; 3 CF: MD. Esp. Medicina Familiar. Universidad El Bosque. Bogotá, Colombia. cfuentese@unbosque.edu.co Universidad El Bosque Universidad El Bosque Bogotá Colombia cfuentese@unbosque.edu.co; 4 JT: MD. Esp. Psiquiatría. Epidemiol. M. Sc. Ensayos Clínicos. Universidad El Bosque. Bogotá, Colombia. toledojose@unbosque.edu.co Universidad El Bosque Universidad El Bosque Bogotá Colombia toledojose@unbosque.edu.co

**Keywords:** Medicina familiar, burnout, Colombia *(fuente: DeCS, BIREME)*, Family medicine, burnout, Colombia *(source: MeSH, NLM)*

## Abstract

**Objetivo:**

Determinar la prevalencia y los factores asociados a la presencia de síndrome de *burnout* en los médicos familiares en ejercicio en Colombia en el año 2021.

**Métodos:**

Se desarrolló un estudio transversal analítico en el cual, en un momento dado en el tiempo, se tomaron datos de una serie de sujetos (profesionales en Medicina Familiar) y se realizó en el mismo momento una encuesta y el cuestionario de Maslach. Se describió esta población. A continuación, se usó el cuestionario citado previamente, se dividió el grupo en personas con el síndrome de *burnout* y sin dicho síndrome, y con esta información se procedió a construir tablas de 2x2, donde se obtuvieron los respectivos OR para cada factor de riesgo o protector. Se obtuvo una prevalencia de punto. Para hacer estimación de parámetros se calcularon los intervalos de confianza y se probó cada una de las hipótesis con una p de 0,05.

**Resultados:**

Se analizó un total de 206 encuestas. Se encontró una prevalencia de síndrome de burnout de 48% (IC 95% 47,54-48,46), la cual abarcó una muestra que comprende 33 ciudades diferentes en Colombia, y se halló como factor de riesgo estadísticamente significativo el encontrarse trabajando con la modalidad de contrato por orden de prestación de servicios, con un OR de 2,11 (IC 95% 1,19-3,72 p=0,007).

**Conclusión:**

Este estudio muestra que uno de cada dos médicos familiares en Colombia presenta síndrome de burnout, lo que refleja posibles dificultades en su labor que requieren ser evaluadas e intervenidas para mitigar esta condición.

## Abstract (en)

**Objective:**

To determine the prevalence and factors associated with the presence of burnout syndrome in practicing family physicians in Colombia in the year 2021.

**Methods:**

An analytical cross-sectional study was developed in which, at a given moment in time, data was taken from a series of subjects (Family Medicine professionals) and a survey and the Maslach questionnaire were carried out at the same time. This population was described. Next, the previously mentioned questionnaire was used, the group was divided into people with Burnout Syndrome and those without Burnout Syndrome, and with this information 2x2 tables were constructed, where the respective OR were obtained for each risk factor or protective. A point prevalence was obtained. To estimate parameters, confidence intervals were calculated and each of the hypotheses was tested with a p of 0.05.

**Results:**

A total of 206 surveys were analyzed. A prevalence of Burnout syndrome of 48% was found (CI 95% 47.54-48.46), which covered a sample that includes 33 different cities in Colombia, and it was found as a statistically significant risk factor to be working with the modality of contract by order of provision of services with an OR of 2.11 (CI 95% 1.19-3.72 p=0.007).

**Conclusion:**

This study shows that 1 out of 2 family physicians in Colombia have Burnout syndrome, reflecting possible difficulties in their work that require evaluation and intervention to mitigate this condition.

El síndrome de *burnout* se define en el CIE-11 como un resultado del estrés crónico en el lugar de trabajo que no se ha manejado con éxito. Se caracteriza por tres dimensiones: 1) sentimientos de falta de energía o agotamiento; 2) aumento de la distancia mental con respecto al trabajo, o sentimientos negativos o cínicos con respecto a este; y 3) eficacia profesional reducida 1. Es un factor de riesgo laboral y de salud 2 que altera los ámbitos psicosociales del individuo que lo padece y tiene repercusiones sobre el grupo cercano. Este impacto lo lleva a ser considerado un problema de salud pública, con tanta importancia que ha sido vigilado por la Organización Mundial de la Salud (OMS) 3.

La Ley 1438 del 19 de enero de 2011, que adopta la estrategia de atención primaria en salud (APS) como eje integral del sistema general de seguridad social en salud, define la APS como "la estrategia de coordinación intersectorial que permite la atención integral e integrada, desde la salud pública, la promoción de la salud, la prevención de la enfermedad, el diagnóstico, el tratamiento, la rehabilitación del paciente en todos los niveles de complejidad a fin de garantizar un mayor nivel de bienestar en los usuarios, sin perjuicio de las competencias legales de cada uno de los actores del Sistema General de Seguridad Social en Salud" (4, p. 7). Además, en el 2016 con la aparición del Modelo Integral de Atención en Salud (MIAS), para la prestación de servicios en salud se requiere crear equipos interprofesionales, idealmente con la participación de especialistas de medicina familiar, con el fin de brindar, de forma integral, un correcto cuidado de la salud y bienestar de la comunidad. Este profesional se encuentra inmerso en un sinnúmero de funciones, por lo cual es necesario que cuente con un ambiente saludable donde ejerza su labor de la mejor forma.

La prevalencia del síndrome de *burnout* se ha estudiado a nivel global y se han encontrado valores diversos: en África, sobre el 81% 5; en Asia, un rango entre 40 y 60% 6; en Europa, entre 22 y 58,6% 7,8; en Norteamérica, 54% 9; y en Latinoamérica, entre 14 y 80% 10,11. En Colombia, la encuesta de situación laboral para los profesionales de la salud 2019 revela un panorama comprometedor para este sector, dado que se evidencia que el 33% de los médicos especialistas debe trabajar entre 48 y 66 horas a la semana, el 56% tiene una contratación por prestación de servicios, el 71% no ha recibido a tiempo el pago de salarios, y el 77% de los profesionales de la salud incluidos en la encuesta (médicos rurales, generales y especialistas) se siente inconforme con su salario, piensa que está siendo explotado y no ve compensado el esfuerzo y la millonaria inversión que tuvo que hacer para costear los estudios profesionales 12.

En Colombia, sobre el síndrome de *burnout* se han desarrollado estudios enfocados en médicos y estudiantes de residencias médico-quirúrgicas y otros personales de la salud, en los que se reportan valores entre el 2,2 y el 29,3% 13,14. En Medicina Familiar, algunas tesis evalúan este síndrome en residentes de la especialidad en Colombia con valores de 34,93 y 58% 15-17. En Colombia, al momento de la revisión bibliográfica, no existen publicaciones basadas en determinar la prevalencia de *burnout* en médicos familiares en ejercicio de la profesión, que se supone alta según informes regionales para los diferentes profesionales en salud 18-24. En este estudio se muestra un avance para determinar la prevalencia del mencionado síndrome, y algunas asociaciones, de acuerdo con la literatura, predisponen a su aparición.

Se trató de un estudio transversal analítico. Para su desarrollo se aplicó una encuesta con un cuestionario sociodemográfico con base en la literatura disponible, y para la identificación del síndrome de *burnout* se usó la escala de Maslach (MBI), 25 de manera virtual, mediante el aplicativo Google Forms®, con el objeto de abarcar participantes de diferentes lugares del país y evitar una exposición a riesgos debido a la contingencia por el SARS-COV-2.

Con referencia al síndrome de *burnout* en médicos familiares en ejercicio en Colombia, se encontró una prevalencia del 48% (IC 95% 47,54-48,46) de esta condición, con una proporción en sus tres dimensiones, de manera individual, de un 34% en cuanto a cansancio emocional; despersonalización, 27,2%, y no realización personal, 12,1%. Algunos individuos presentaron dos o más de estas áreas afectadas, así como una asociación estadísticamente significativa con la contratación por OPS con un OR de 2,1 (IC 95% 1,195-3,728 p=0,007).

## MÉTODOS

Este es un estudio observacional descriptivo transversal, en el cual, con la previa aceptación del consentimiento informado, se recolectó información de profesionales de medicina familiar colombianos, o con título convalidado en nuestro país, laboralmente activos en el momento de la aplicación de la encuesta. Se llevó a cabo una estimación de especialistas en Medicina Familiar en ejercicio en Colombia, de 837 profesionales a inicios del 2021, según los datos suministrados en el IX Congreso Nacional de Medicina Familiar y por la Sociedad Colombiana de Medicina Familiar (SOCMEF). Se hizo un cálculo muestral utilizando el paquete EPI-INFO, con intervalo de confianza (IC) del 95% y un margen de error del 5%, con n=290 médicos familiares (aumentando 10% por posibles pérdidas).

El periodo de recolección de encuestas comprendió del 13 de julio 2021 hasta el 11 de noviembre del mismo año. Se obtuvieron 198 encuestas, de las cuales se excluyeron seis, por tratarse de médicos familiares que laboraban fuera de Colombia, y otras tres por tratarse de registros duplicados. Dado que los 189 registros resultantes no cumplían con la muestra calculada inicialmente, se propuso modificar el cálculo muestral. Así, se conservó el intervalo de confianza del 95% y la estimación de médicos familiares de 837, con la modificación del margen de error, ahora de 6%, lo cual implica un n = 203 médicos familiares. Por tanto, se abrió un espacio para reclutar nuevos registros entre el 15 de enero 2022 y el 17 de enero de 2022, y se obtuvieron 17 registros adicionales, para un total de 206 registros, cumpliendo así la muestra calculada por segunda intención.

Se aplicó una encuesta sociodemográfica y el cuestionario Maslach (MBI). Esta última es la herramienta diagnóstica de síndrome de *burnout,* que se encuentra debidamente validada 26, la cual aborda tres subescalas: agotamiento emocional, despersonalización y realización personal. La encuesta sociodemográfica se usó para establecer asociaciones y determinar posibles factores de riesgo (como sexo y lugar de procedencia) y demás variables reportadas en la literatura.

La recolección de datos se llevó a cabo de forma digital, debido a la coyuntura de la pandemia global y para facilitar la participación de profesionales de diferentes lugares del país. Con estos datos se describió esta población tanto con variables numéricas como categóricas. A continuación, usando el cuestionario Maslach, se dividió el grupo en personas con el síndrome de *burnout,* o sin este, y con dicha información se construyeron tablas de 2x2, a partir de las cuales se obtuvieron los respectivos OR para cada factor de riesgo o protector con IC y p=0,05 para probar hipótesis. Al contar con el número de sujetos con síndrome de *burnout* y el total de médicos familiares encuestados, se obtuvo una prevalencia de punto.

Se hizo un análisis descriptivo de todos los datos para las variables numéricas mediante la prueba de Kolmogorov-Smirnov, dado que presentaron una distribución paramétrica y se elaboraron tablas de frecuencia, y se obtuvo media y desviación estándar. Para las variables categóricas se elaboraron tablas de frecuencia y se encontraron proporciones. Se obtuvo la proporción de prevalencia de *burnout.* Todos estos datos se entregaron con prueba de hipótesis (p=0,05) y los respectivos intervalos de confianza del 95%.

## RESULTADOS

Una vez realizado el cálculo del tamaño muestral (203 individuos), se aplicó la encuesta y se llegó a un total de 215 registros, los cuales al ser filtrados (se eliminaron tres por duplicidad y seis por laborar fuera de Colombia) arrojaron una muestra final de 206. Con estos formularios se hizo el análisis estadístico de los datos recolectados y se encontró una prevalencia de síndrome de *burnout* del 48% (IC 95% 47,54-48,46) para los médicos familiares participantes. La muestra poblacional abarca profesionales que trabajan principalmente en la ciudad de Bogotá, sin embargo, comprende 33 ciudades diferentes (Tabla 1), distribuidas sobre el territorio colombiano, y dos profesionales que no indicaron su lugar de residencia (no obstante, completaron a cabalidad el resto del cuestionario). En cuanto a edades, se encontraron entre los 26 y los 67 años, con una media de 39 años y una moda de 32 años, y principalmente participaron mujeres. Con referencia a variables familiares, en estado civil se encontró que más del 64% tiene una unión marital y el 42% no tiene hijos.

**Tabla 1:** Ciudad de residencia

Ciudad de residencia	Numero de datos
Armenia	2
Bahía Málaga	1
Barranquilla	2
Bogotá	124
Bucaramanga	14
Cajicá	2
Cali	13
Cartagena	1
Chía	3
Chiquinquirá	1
Cundinamarca	1
Duitama	1
No registra	2
Facatativá	1
Florencia	1
Funza	3
Fusagasugá	2
Ibagué	1
Medellín	3
Moniquirá	1
Montería	1
Neiva	1
Palmira	1
Pasto	1
Pereira	4
Piedecuesta	1
Pitalito	2
Popayán	5
Sogamoso	1
Tunja	5
Villavicencio	1
Villeta	1
Yopal	2
Yumbo	1

Al aproximarse al ámbito laboral, el 75,7% cuenta con una experiencia como especialistas inferior a 10 años; el 55% tiene un solo empleo. Las características más frecuentemente encontradas con respecto a la asignación salarial fueron de 5 a 10 SMMLV, así como una carga horaria entre 37 y 48 horas semanales. En esta misma línea, el 64% trabaja en consulta externa de medicina familiar; el tipo de contratación más reportado por los participantes fue el de contrato por prestación de servicios (OPS), con un 39,3%, seguido del contrato a término indefinido con 35,4%. Y, por último, a nivel sociodemográfico, se encontró que al 16% de los encuestados se les adeuda pagos por su labor (Tabla 2).

**Tabla 2:** Resultados obtenidos

Datos obtenidos	n	%
Edad		
26-30	16	7,77
31-35	64	31,07
36-40	47	22,82
41-45	38	18,45
46-50	16	7,77
51-55	10	4,85
56-60	5	2,43
61-65	9	4,37
66-70	1	0,49
Género		
Femenino	132	64,08
Estado Civil		
Casado(a)	101	49,03
Divorciado(a)	9	4,37
Soltero(a)	64	31,07
Unión libre	32	15,53
Número de hijos		
0	88	42,72
1	51	24,76
2	58	28,16
3	7	3,40
4	2	0,97
Tiempo de ejercicio como especialista en medicina familiar		
Menos de 1 año	40	19,42
Mayor a 1 año y menos de 5 años	67	32,52
Más de 5 años y menos 10 años	49	23,79
Más de 10 años y menos de 15 años	18	8,74
Mayor de 15 años	32	15,53
Número de trabajos que desempeña actualmente		
1	114	55,34
2	72	34,95
3	17	8,25
4	2	0,97
5 o más	1	0,49
Número de SMMLV recibidos mensualmente (2021)		
1 a 5 SMMLV ($908 526-$4 542 630)	26	12,62
Entre 5 y 10 SMMLV ($4 542 631-$9 085 260)	94	45,63
Entre 10 y 15 SMMLV ($9 085 261- $13 627 890)	72	34,95
Más de 15 SMMLV (Más de $13 627 891)	14	6,80
Suma de horas laboradas durante la última semana		
Hasta 36 horas semanales	47	22,82
De 37 horas a 48 horas semanales	97	47,09
Más de 49 horas semanales	62	30,10
Cargo que desempeña en su lugar de trabajo		
Academia (enseñanza, educación)	21	10,19
Administrativo	24	11,65
Consulta externa	132	64,08
Domiciliario	1	0,49
Hospitalización	8	3,88
Urgencias	20	9,71
Tipo de contratación		
Cooperativa	1	0,49
Obra labor	7	3,40
Particular	1	0,49
Prestación de servicios	81	39,32
Salario integral	2	0,97
Termino fijo	41	19,90
Termino indefinido	73	35,44
Se le debe algún salario actualmente		
Sí	35	16,99

n= número de respuestas, SMMLV= salarios mínimos mensuales legales vigentes.

Adicionalmente, al hacer un análisis de tablas de 2x2, comparando la presencia de síndrome de *burnout* con las diferentes variables sociodemográficas, se obtuvo una relación estadísticamente significativa entre el padecer esta condición y encontrarse trabajando con la modalidad de orden de prestación de servicios (OPS) con un OR de 2,11 (IC 95% 1,19-3,72 p=0,007). En relación con otros factores, no se encontró significancia estadística (Tabla 3).

**Tabla 3:** Factores asociados

**Factores asociados a *burnout***	OR	p
Contrato OPS	2,11 (IC 95% 1,19-3,72)	0,007
Deuda de salario	1,78 (IC 95% 0,85-3,74)	0,08
Más de 48 horas semanales laboradas	1,47 (IC 95% 0,81-268)	0,13
Trabajar en urgencias	1,36 (IC 95% 0,53-3,43)	0,33
Más de cinco años de labor	1,2 (IC 95% 0,7-2,11)	0,28
Más de un trabajo	1,15 (IC 95% 0,66-1,99)	0,35
Trabajar en consulta externa	1,14 (IC 95% 0,64-2,01)	0,37
Unión Marital	1,08 (IC 95% 0,61-1,91)	0,45
Tener hijos	0,87 (IC 95% 0,5-1,51)	0,36
Menos de cinco años de labor	0,64 (IC 95% 0,37-1,12)	0,08
Más de 10 SMMLV	0,58 (IC 95% 0,3-1,11)	0,07
Menos de 10 SMMLV	0,6 (IC 95% 0,27-1,3)	0,13

OR=odds ratio, OPS= orden de prestación de servicios, IC= intervalo de confianza, SMMLV= salarios mínimos mensuales legales vigentes.

Una vez realizado el análisis de regresión logística se corroboró el dato obtenido con el análisis previo (Tabla 4). Al desglosar el síndrome de *burnout* en sus tres dimensiones, el cansancio emocional se ve reflejado en el 26,1% de los participantes, la despersonalización se encuentra en el 20,9% de los encuestados y, por último, la realización personal se ve afectada en el 9,3%.

**Tabla 4:** Análisis de regresión logística

Variable	B	Error estándar	Wald	Significancia	Exp(B)
Contratación por OPS	0,952	0,338	7,906	0,005	2,590

OPS= orden de prestación de servicios.

## DISCUSIÓN

La elaboración de este estudio mostró varios tópicos que merecen ser discutidos. Esta investigación arrojó una prevalencia del 48% (IC 95% 47,54-48,46) de síndrome de *burnout* en la muestra analizada, muy por debajo de la reportada en África, del 81% 5; similar al rango en Asia, de 40-60% 6, y de Europa entre 22% y 58,6% 7,8, y por debajo del encontrado en Norteamérica del 54% 9. En Sudamérica, la prevalencia de *burnout* se sitúa por debajo de la encontrada en este estudio, superada solo por la reportada en Perú con el 80%. 24 Estas diferencias quizás se deban a las características propias de cada país o región.

En Colombia, la prevalencia de síndrome de *burnout* en médicos familiares es superior a la encontrada en estudios que analizaron personal de la salud en general, de entre 2,2 y 29,3% 13,14, quizá debido a factores como los mencionados por otros autores 27; por ejemplo, desconocimiento de la especialidad por el público en general, pocas vacantes y bajos salarios, entre otros, en concordancia con lo encontrado en estudios en residentes de Medicina Familiar donde la prevalecía oscila entre 34,93 y 58% 15-17.

Esta prevalencia es realmente significativa, pues indica que casi uno de cada dos médicos familiares puede estar presentando esta condición. En este mismo sentido, al analizar el *burnout* en sus dimensiones y compararlo con estudios previos, encontramos una prevalencia de agotamiento emocional del 34%, lo que dista bastante del 94,6% 28 reportado en otro estudio, sin embargo, hay que tener en cuenta que en nuestro estudio se tomó padecer el síndrome de *burnout* y no solamente estar en riesgo de presentarlo como en el estudio citado. En despersonalización y realización personal, el 27,2% y el 12,1%, respectivamente, mientras que para la misma publicación estas dos categorías tuvieron un valor de 53,6% 28.

En cuanto a los datos sociodemográficos, vale la pena destacar la distribución de la población, cuya mayoría son mujeres, concordante con la proporción creciente de este género en las ciencias de la salud. A su vez, los adultos jóvenes comprenden el mayor porcentaje de médicos familiares encuestados, y su mayoría se encuentra en unión marital, en tanto que un poco más de la mitad tiene al menos un hijo. Al observar la distribución de los participantes en el territorio nacional, encontramos apoyo en todos los puntos cardinales del país, a pesar de que la mayor participación se logró en Bogotá, donde se concentra la mayor proporción de médicos familiares en el país, según otros estudios 27.

Al analizar los factores correspondientes al ejercicio de la especialidad, el 50% de los encuestados tiene una experiencia laboral como médicos familiares de entre uno y diez años, con un predominio entre uno y cinco años, lo que representa el 32,5%. Lo anterior concuerda con el hecho de que esta especialidad es relativamente reciente en nuestro país, pues los primeros graduandos iniciaron su labor en 1984 en la Universidad del Valle y la primera promoción, en universidades como El Bosque, se graduó en el 2002. Otro factor llamativo fue que el 55% tiene solo un trabajo al momento de su participación, lo cual podría estar en relación con lo mencionado por otros autores 27, que hace referencia a la escasa oferta laboral para esta especialidad médica en Colombia.

Al contrastar los datos obtenidos a nivel profesional con la encuesta de situación laboral para los profesionales de la salud 2019 12, encontramos similitud en el número de horas laboradas a la semana, con un 30,1% vs. un 33% en la encuesta del 2019; y el tipo de contratación por OPS fue el más prevalente, con un 39% vs. un 56% en 2019. Para lo referente al adeudamiento de salarios, se evidencia solo un 16,9% en nuestro estudio, contra un 71% en todos los profesionales en salud en 2019. Por último, a nivel ocupacional, los médicos familiares se desempeñan principalmente en cargos de consulta externa y en menor medida en cargos administrativos, académicos, en urgencias u hospitalización.

Si bien se incluyeron diversas variables sociodemográficas en el estudio, acorde a lo reportado en la literatura, al practicar el análisis multivariado se encontró asociación estadísticamente significativa solo con el tipo de contratación por OPS, con un OR de 2,1 (IC 95% 1,195-3,728 p=0,007), concordante con otros estudios hechos en Colombia 13. Esto quizás en relación con las dificultades de empleabilidad y la precarización laboral que en general se observa en el área de la salud en el país, aún más con este tipo de contratación que no brinda estabilidad laboral ya que el profesional se encuentra supeditado a las voluntades del empleador de continuar o no con su contrato. También este tipo de relación ocupacional cohíbe al médico del disfrute de derechos de bienestar como las vacaciones, las primas o las cesantías.

El principal inconveniente en el desarrollo del estudio fue la baja participación de los médicos familiares en la realización de la encuesta, lo que conllevó un ajuste en el tamaño muestral y un aumento leve del error (del 5 al 6%). Por otra parte, este es un estudio original que aborda una población en la cual no se han desarrollado estudios sobre síndrome de *burnout* previamente, lo cual genera un nuevo conocimiento y abre puertas para nuevos estudios que profundicen el conocimiento de esta condición. Este estudio sienta las bases para llevar a cabo propuestas de investigación para conocer otros factores asociados a este síndrome o por qué el tipo de contratación afecta el agotamiento de los profesionales, entre otros. También sería de utilidad evaluar los retos que conllevó la contingencia sanitaria por el SARS-COV-2; para conocer el impacto de la pandemia en la labor del médico familiar, como de otros profesionales, se requieren estudios adicionales, quizá al finalizar dicha eventualidad mundial.

Aunque no se encontraron otras asociaciones de riesgo significativas, es de anotar que una prevalencia del 48% de síndrome de *burnout* en los médicos familiares en Colombia no es un valor despreciable. Esta condición puede impactar significativamente el quehacer profesional y personal de los médicos familiares, quienes están llamados a ser uno de los pilares fundamentales del sistema de salud colombiano. Por esto, se deben buscar las herramientas necesarias por parte de los empleadores e incluso del sistema de salud mismo para prevenir la aparición de esta condición en más profesionales de la salud, y en quienes lo presentan dar solución a las problemáticas que los llevaron a este estado. Una de estas herramientas podría ser la formulación de una ruta de atención en salud para esta condición que incluya su diagnóstico oportuno y estrategias de atención integral ♣
